# Child Body Mass Index and Health Care Costs in England

**DOI:** 10.1001/jamanetworkopen.2025.37560

**Published:** 2025-10-14

**Authors:** Olu Onyimadu, Alison Hayes, Patrick Fahr, Cynthia W. Drakesmith, Nerys M. Astbury, Mara Violato, Stavros Petrou

**Affiliations:** 1 Nuffield Department of Primary Care Health Sciences, University of Oxford, Oxford, United Kingdom; 2 School of Public Health, Faculty of Medicine and Health, University of Sydney, Sydney, New South Wales, Australia; 3 Nuffield Department of Population Health, University of Oxford, Oxford, United Kingdom

## Abstract

**Importance:**

In England, 27% of children aged 2 to 15 years live with overweight or obesity. However, national estimates of excess health care costs attributable to unhealthy weight status are lacking.

**Objective:**

To estimate health care costs associated with childhood weight status, stratified by sex, age, and ethnicity, in England.

**Design, Setting, and Participants:**

This retrospective cross-sectional study used 2014 to 2020 Clinical Practice Research Datalink Aurum data linked with Hospital Episode Statistics at general practices in England. Children aged 2 to 15 years with at least 1 body mass index (BMI) measurement recorded during a general practice visit between January 1, 2015, and March 30, 2019, for whom primary care and linked Hospital Episode Statistics data were available were studied. The analysis was conducted from December 11, 2023, to March 17, 2025.

**Exposure:**

BMI-derived weight status categories.

**Main Outcomes and Measures:**

Total health care costs were analyzed for the 1-year period before and after BMI measurement. Attributable health care costs were estimated using 2-part generalized linear models, adjusted for sex, age, ethnicity, socioeconomic status, and geographic region.

**Results:**

The study included the health care records of 268 231 unique children (mean [SD] age, 6.82 [2.66] years; 148 230 [55%] male; 4554 [2%] Bangladeshi, 16 708 [6%] Black, 7778 [3%] Chinese or other Asian, 7665 [3%] Indian, 10 875 [4%] Pakistani, 169 299 [63%] White, 10 886 [4%] multiracial, and 40 466 [15%] other or unknown). Before BMI measurement, health care costs were higher for children with overweight, obesity, and severe obesity but lower for children with underweight compared with children with healthy weight. After BMI measurement, costs were higher in children with underweight, overweight, obesity, and severe obesity compared with children with healthy weight. Severe obesity had the highest mean annual excess costs at £190 (95% CI, £77-£302) (US $274 [95% CI, US $111-US $435]) followed by underweight at £164 (95% CI, £69-£260) (US $236 [95% CI, US $99-US $374]). Health care costs varied by sex, age group, and ethnicity, although interactions were limited to specific BMI categories and covariate levels. The excess annual national health care cost attributable to overweight and obesity was £0.27 billion (95% CI, £0.12 billion to £0.43 billion) (US $0.39 billion [95% CI, US $0.17 billion to US $0.62 billion]).

**Conclusions and Relevance:**

In this cross-sectional study of children, health care costs were higher for nearly all unhealthy weight categories compared with healthy weight BMI measurement. These findings support the economic case for preventive and therapeutic interventions targeting unhealthy childhood weight and offer cost estimates for use in cost-effectiveness analyses.

## Key Points

**Question:**

What is the health care cost burden attributable to unhealthy weight status among children in England?

**Findings:**

In this cross-sectional study of 268 231 children accessing health care in England, underweight, overweight, obesity, and severe obesity were associated with higher health care costs compared with healthy weight 1 year after body mass index assessment. For underweight and severe obesity, these excess annual costs were £164 (US $236) and £190 (US $274), respectively.

**Meaning:**

Cost estimates generated from this research can potentially inform clinical and budgetary service planning and serve as parameter inputs for studies evaluating the cost-effectiveness of interventions targeting childhood unhealthy weight.

## Teaser

This cross-sectional study estimates the health care costs associated with childhood weight status among children aged 2 to 15 years accessing health care in England.

## Introduction

The UK ranks among the top 10 countries in Europe and the Organisation for Economic Co-operation and Development (OECD) for obesity prevalence among children and adults.^1,2^ In England, where approximately 84% of the UK population resides, 27% of children aged 2 to 15 years had overweight or obesity in 2022.^3,4^ Childhood excess weight (overweight or obesity) presents a critical public health challenge in high-income and emerging economies.^5^ In the short term, children with excess weight are at increased risk of mental health disorders, type 2 diabetes,^6^ asthma,^7^ and poorer educational outcomes.^5,8^ In the long term, childhood excess weight significantly raises the risk of adult obesity,^9,10,11^ which is associated with chronic conditions, including type 2 diabetes, cardiovascular disease, and several types of cancer,^12^ burdening individuals, the health care system, and the wider economy.^13^

Estimating health care costs associated with childhood weight status is essential for informing policy decisions on whether interventions that mitigate this challenge are cost-effective. However, evidence on these costs remains limited, particularly in the UK, where no national estimates on the economic burden of childhood unhealthy weight derived from electronic health record data exist.^14,15^ Related UK research is scarce, often focusing on narrow age groups and excluding underserved populations.^16,17^ Few studies explore health care costs of childhood underweight^18,19,20^ or disaggregate estimates by ethnicity, often using broad categories, potentially obscuring subgroup variations.^17,20^ Furthermore, limited research considers the timing of body mass index (BMI) measurement when estimating annual health care costs, leaving a crucial gap. By analyzing costs in the year before and after BMI measurement, we aimed to provide a clearer temporal context and a more comprehensive understanding of cost patterns than previous studies. Insights derived from UK-based studies are pivotal in addressing knowledge gaps regarding the economic impact of childhood excess weight, given the UK’s high prevalence of childhood obesity compared with other European and OECD nations.^1,2^ This study aimed to estimate the primary and secondary health care costs associated with childhood weight status among children aged 2 to 15 years.

## Methods

### Data Sources

We used the Clinical Practice Research Datalink (CPRD) Aurum, covering 18 million currently registered patients^21,22,23^ and containing pseudoanonymized, routinely recorded consultations, prescriptions, and diagnostic test results from general practices.^23,24^ This study was approved by the CPRD Research Data Governance process; the protocol is publicly available on the CPRD website.^61^ CPRD-registered patients are representative of the UK’s population in terms of patient-level socioeconomic status, although slightly overrepresented in deprived areas.^25^ We used CPRD records linked to 3 Hospital Episode Statistics (HES) databases: admitted patient care (inpatient), outpatient hospital records, and accident and emergency datasets.^26,27^ HES captures up to 98% of hospital activity (secondary care) in England, offering comprehensive administrative data to support the analysis of health care use.^28^ No informed consent was required because the study did not directly interact with patients. The study followed the Strengthening the Reporting of Observational Studies in Epidemiology (STROBE) reporting guideline.^30^

### Study Population 

Records of children aged 2 to 18 years who were resident in England and registered with a CPRD Aurum practice were eligible for the study if there was at least 1 BMI measurement recorded between January 1, 2015, and March 30, 2019, and their primary care records could be linked to HES data. Because the main focus is on costs and the costing methods before 2015 was not comparable to after 2015,^29^ we restricted our sample to records from 2015 onward to minimize potential biases. To assess the representativeness of our sample with recorded BMIs, we compared the BMI category distribution with National Child Measurement Programme surveillance data^4^ (eFigures 1-2 and eTable 1 in Supplement 1).

### Study Design

Using a retrospective cross-sectional design, we analyzed primary and secondary health care use and costs for 2 periods: 1 year before and 1 year after the date of BMI measurement. Data cleaning and processing are summarized in eFigure 3 and eTables 2 to 7 in Supplement 1. If a child had multiple BMI measurements within a year, the last recorded measurement was selected as the index date. Health care resource use was assessed in the 12 months before and the 12 months after this date. A sensitivity analysis was conducted using the first BMI measurement each year.

### Exposure: Weight Status Category

To determine exposure levels (weight status categories), BMI (calculated as weight in kilograms divided by height in meters squared) was calculated from contemporaneous height and weight measurements and converted into SD scores (BMI *z* scores) using the British Growth Reference (UK90), which provides age-adjusted and sex-specific *z* scores.^31^ These values were categorized according to UK population monitoring thresholds: 2nd centile or less (underweight), greater than 2nd to less than 85th centile (healthy weight), 85th or greater to less than 95th centile (overweight), 95th or greater to less than 99.6th centile (obesity), and 99.6th centile or greater (severe obesity).^32^ Only the month of birth was provided; thus, it was assumed that all participants were born on the 15th day of their birth month.

### Costing

Resource inputs were valued using the Unit Costs of Health and Social Care compendium^33^ for primary care (eTables 2-4 in Supplement 1) and the National Cost Collection Data schedules^34^ for secondary care (eTables 5-7 in Supplement 1). Cost outcomes were adjusted using the Campbell & Cochrane Economics Methods Group–Evidence for Policy and Practice Information–Centre Cost Converter, with all costs expressed in 2022 Great British pounds and US dollars.^35^

### Covariates

Covariate selection was guided by relevant literature and data availability.^20,36,37,38,39^ The final model adjusted for child age group, sex, ethnicity, geographic region, and area-level deprivation using the English Index of Multiple Deprivation.^40^ Ethnicity was derived from CPRD and HES records, reflecting patient self-report using standard National Health Service (NHS) classification categories (including Bangladeshi, Black, Chinese or other Asian, Indian, mixed, Pakistani, White, or other [coded as any other ethnic group] or unknown. We collected data on ethnicity to assess potential differences in outcomes across demographic groups.

### Statistical Analysis

We used a 2-part generalized linear model (GLM) to examine the association between weight status and total health care costs, accounting for the many zero-cost cases and the right-skewed distribution of costs.^41,42^ A logit model predicted the probability of health care use, followed by a GLM with log link and γ-distribution to estimate mean annual costs for nonzero users. Marginal estimates derived from the Stata margins command average both stages of the 2-part model and are expressed as mean absolute costs by BMI category and covariates. We used robust SEs, widely accepted for large-sample generalized linear and 2-part models, with 95% CIs derived via the Δ method.^42^ Clustered regression analyses at the patient level accounted for within-patient correlation.

The selection of interaction terms relied on results from the combined log pseudolikelihood, Akaike information criterion (AIC), bayesian information criterion (BIC), and deviance estimates.^43,44,45^ Overall model selection was informed by diagnostic tests (ie, Park test for variance structure and Box-Cox regression for link function evaluation)^46^ complemented, when necessary, by AIC and BIC tests (eTables 8 and 9 in Supplement 1).^43,44,45^ The analysis included all observations unless cost data errors, implausible BMI values, or miscoded procedures were identified. This approach preserved the full distribution of health care costs.^39,47^

We generated unadjusted estimates (model 1) and then adjusted both components of the 2-part model for covariates (model 2). In model 3, interaction terms between BMI category and covariates were added through an iterative, theory-informed process guided by prior literature,^20^ retaining only those that improved AIC and BIC statistics, or deviance without compromising parsimony (eTables 10 and 11 in Supplement 1). The analysis was conducted from December 11, 2023, to March 17, 2025, using Stata, version 17 statistical software (StataCorp).^48^

The total annual national health care costs attributable to childhood excess weight were estimated based on population data from the Office for National Statistics,^49^ the Royal College of Paediatrics and Child Health,^50^ and NHS England,^51^ reporting on 2.5 million affected children in England, including 1.22 million with obesity. Adjusted mean incremental costs by weight category were applied to these estimates. This method aligns with prior studies on BMI-related population costs.^18,20,52^

## Results

Our study included the electronic health care records of 268 231 unique children (mean [SD] age, 6.82 [2.66] years; 148 230 [55%] male and 120 001 [45%] female; 4554 [2%] Bangladeshi, 16 708 [6%] Black, 7778 [3%] Chinese or other Asian, 7665 [3%] Indian, 10 875 [4%] Pakistani, 169 299 [63%] White, 10 886 [4%] multiracial, and 40 466 [15%] other or unknown), with 180 438 children (67%) categorized as having a healthy weight (Table 1). Boys outnumbered girls, consistent with a prior large-scale UK cohort study.^53^ Weight status category distributions were similar between sexes but varied by age, with higher prevalence of obesity and severe obesity in older children (Table 1). BMI distributions in our cohort closely aligned with the National Child Measurement Programme, with only modestly higher obesity and underweight rates, as expected in primary care, where BMI is measured more often (eResults in Supplement 1).

**Table 1.** Demographic Characteristics of the Study Population and Costs by BMI Category Among 268 231 Unique Children Aged 2 to 15 Years^a^

Characteristic	No. (%) of study participants^b^				
	≤2nd Centile (underweight) (n = 11 002)	>2nd and <85th Centile (healthy weight) (n = 180 438)	≥85th and <95th Centile (overweight) (n = 30 950)	≥95th and <99.6th Centile (obesity) (n = 30 590)	≥99.6th Centile (severe obesity) (n = 15 251)
Sex					
Male	6280 (4)	98 367 (66)	17 221 (12)	17 167 (12)	9195 (6)
Female	4722 (4)	82 071 (68)	13 729 (11)	13 423 (11)	6056 (5)
Age group, y					
2-3	2333 (5)	34 289 (71)	5958 (12)	4113 (8)	1714 (4)
4-5	2997 (5)	44 258 (72)	6833 (11)	5012 (8)	2714 (4)
6-7	2510 (4)	44 196 (70)	6509 (10)	6290 (10)	3760 (6)
8-9	1819 (3)	36 100 (63)	6775 (12)	8569 (15)	4025 (7)
10-11	1213 (4)	19 488 (58)	4352 (13)	5947 (18)	2703 (8)
12-13	111 (3)	1808 (56)	454 (14)	573 (18)	291 (9)
14-15	19 (4)	299 (58)	69 (13)	86 (17)	44 (9)
Ethnicity					
Bangladeshi	359 (8)	2842 (62)	420 (9)	617 (14)	316 (7)
Black	632 (4)	10 173 (61)	2165 (13)	2349 (14)	1389 (8)
Chinese or other Asian	551 (7)	5236 (67)	749 (10)	836 (11)	406 (5)
Indian	845 (11)	5074 (66)	643 (8)	759 (10)	344 (4)
Pakistani	919 (8)	7091 (65)	987 (9)	1168 (11)	710 (7)
White	5132 (3)	116 492 (69)	20 167 (12)	18 608 (11)	8900 (5)
Multiracial	430 (4)	7073 (65)	1297 (12)	1299 (12)	787 (7)
Other or unknown^c^	2134 (5)	26 457 (65)	4522 (11)	4954 (12)	2399 (6)
Index of Multiple Deprivation, quintile					
Fifth (lowest level of deprivation)	1635 (4)	30 137 (73)	4483 (11)	3576 (9)	1370 (3)
Fourth	1714 (4)	30 244 (71)	4849 (11)	4322 (10)	1758 (4)
Third	1922 (4)	32 447 (68)	5405 (11)	5353 (11)	2435 (5)
Second	2706 (4)	40 402 (65)	7265 (12)	7612 (12)	3984 (6)
First (highest level of deprivation)	3025 (4)	47 208 (63)	8948 (12)	9727 (13)	5704 (8)
Region in England					
London	3466 (5)	42 218 (65)	7059 (11)	7800 (12)	4101 (6)
Northeast	234 (3)	6117 (66)	1175 (13)	1147 (12)	629 (7)
Northwest	1654 (4)	30 381 (67)	5541 (12)	5400 (12)	2601 (6)
Yorkshire and The Humber	276 (3)	6483 (68)	1140 (12)	1148 (12)	538 (6)
East Midlands	185 (4)	3629 (69)	606 (12)	558 (11)	274 (5)
West Midlands	1809 (4)	28 354 (65)	5249 (12)	5287 (12)	2792 (6)
East of England	434 (3)	9029 (73)	1353 (11)	1167 (9)	450 (4)
Southeast	1754 (4)	30 115 (70)	4789 (11)	4423 (10)	2125 (5)
Southwest	1079 (3)	21 968 (70)	3584 (11)	3255 (10)	1548 (5)
Missing	111 (3)	2144 (65)	454 (14)	405 (12)	193 (6)
Costs, mean (SD), £^b^^,^^d^					
Primary care costs (the year before index BMI record)	318 (1419)	299 (1039)	336 (4993)	316 (847)	310 (970)
Consultations	126 (173)	125 (173)	125 (174)	125 (173)	131 (180)
Prescriptions	149 (1384)	121 (989)	151 (4981)	126 (773)	118 (914)
Tests and investigations	44 (128)	53 (156)	60 (178)	65 (183)	61 (171)
Primary care costs (the year after index BMI record)	404 (1154)	366 (954)	380 (821)	454 (5514)	393 (893)
Consultations	156 (170)	128 (158)	124 (154)	131 (158)	146 (172)
Prescriptions	162 (1104)	127 (892)	129 (737)	182 (5503)	129 (819)
Tests and investigations	86 (184)	112 (243)	127 (269)	141 (280)	118 (244)
Secondary care costs (the year before index BMI record)	924 (3661)	991 (3839)	1093 (4317)	1036 (4073)	1083 (5318)
Admitted patient care	441 (3004)	458 (3126)	513 (3590)	474 (3413)	479 (3895)
Outpatient care	388 (1043)	432 (1170)	470 (1226)	455 (1183)	493 (1626)
Unintentional injury and emergency care	94 (227)	102 (231)	109 (239)	108 (237)	111 (331)
Secondary care costs (the year after index BMI record)	1059 (3932)	981 (3901)	1046 (3830)	1021 (3921)	1131 (6614)
Admitted patient care	481 (3307)	392 (3271)	409 (3040)	396 (3342)	463 (4968)
Outpatient care	496 (1129)	506 (1154)	546 (1253)	531 (1200)	566 (1826)
Unintentional injury and emergency care	83 (208)	83 (202)	91 (213)	95 (235)	102 (385)
Total health care costs (the year before index BMI record)	1242 (4220)	1290 (4202)	1429 (6831)	1353 (4380)	1393 (5589)
Total health care costs (the year after index BMI record)	1464 (4374)	1347 (4218)	1426 (4112)	1475 (6885)	1524 (6794)

Abbreviation: BMI, body mass index (calculated as weight in kilograms divided by height in meters squared).

^a^ Data from Clinical Practice Research Datalink–Hospital Episode Statistics Electronic Medical Records, 2014 to 2020.

^b^ With respect to the covariates (sex, age distribution, ethnicity, index of multiple deprivation, and region), the values in parentheses are percentages and sum up to 100% across each row. For cost categories (primary care costs, secondary care costs, and total health care costs), the values in parentheses are standard deviations.

^c^ The other ethnicity category represents children coded as “any other ethnic group” in the Clinical Practice Research Datalink dataset.

^d^ To convert to 2022 US dollars, multiply by 1.44.

Regarding model estimates (Table 2; eTables 10 and 11 in Supplement 1), model 3, which incorporated covariates and interaction terms, demonstrated superior performance compared with the unadjusted (model 1) and partially adjusted (model 2) models and was selected as the preferred model for the baseline analysis. Its marginal estimates are reported in Table 3 and Table 4. Absolute predicted annual costs by BMI category are provided in eTable 12 in Supplement 1.

**Table 2.** Mean Marginal Difference in Total Health Care Costs by BMI Category, Sex, Age Group, and Ethnicity Among Children Aged 2 to 15 Years^a^

Covariate	Costs (95% CI), £^b^					
	Year before index BMI record			Year after index BMI record		
	Model 1^c^	Model 2^d^	Model 3^e^	Model 1^c^	Model 2^d^	Model 3^e^
BMI category						
>2nd and <85th Centile (healthy weight)	[Reference]	[Reference]	[Reference]	[Reference]	[Reference]	[Reference]
≤2nd Centile (underweight)	−49 (−130 to 33)	−44 (−122 to 34)	−4 (−95 to 87)	116 (32 to 200)	143 (57 to 229)	164 (69 to 260)
≥85th and <95th Centile (overweight)	139 (60 to 217)	134 (58 to 209)	139 (53 to 225)	78 (28 to 128)	69 (19 to 120)	67 (17 to 116)
≥95th and <99.6th Centile (obesity)	62 (10 to 115)	89 (37 to 142)	113 (54 to 172)	128 (48 to 207)	129 (52 to 206)	141 (68 to 213)
≥99.6th Centile (severe obesity)	103 (12 to 194)	157 (45 to 270)	150 (56 to 243)	177 (67 to 286)	194 (57 to 330)	190 (77 to 302)
Sex						
Male	[Reference]	[Reference]	[Reference]	[Reference]	[Reference]	[Reference]
Female	NA	−77 (−111 to −42)	−79 (−112 to −47)	NA	−76 (−111 to −40)	−76 (−109 to −42)
Age group, y						
2-3	[Reference]	[Reference]	[Reference]	[Reference]	[Reference]	[Reference]
4-5	NA	−425 (−484 to −366)	−411 (−471 to −352)	NA	−268 (−327 to −208)	−251 (−307 to −194)
6-7	NA	−551 (−611 to −491)	−551 (−610 to −491)	NA	−312 (−370 to −255)	−304 (−360 to −248)
8-9	NA	−586 (−648 to −524)	−584 (−643 to −525)	NA	−294 (−350 to −239)	−285 (−338 to −232)
10-11	NA	−391 (−457 to −324)	−380 (−447 to −313)	NA	−88 (−149 to −27)	−70 (−129 to −10)
12-13	NA	−339 (−478 to −200)	−360 (−501 to −219)	NA	58 (−80 to 197)	69 (−79 to 217)
14-15	NA	−176 (−597 to 246)	−186 (−583 to 211)	NA	−188 (−414 to 39)	−158 (−418 to 102)
Ethnicity						
Bangladeshi	NA	−174 (−304 to −45)	−166 (−325 to −8)	NA	−90 (−229 to 48)	−59 (−205 to 88)
Black	NA	−101 (−182 to −20)	−80 (−165 to 6)	NA	−36 (−118 to 45)	−795 (−832 to −758)
Chinese or other Asian	NA	7 (−101 to 116)	9 (−104 to 122)	NA	128 (−114 to 371)	−87 (−178 to 5)
Indian	NA	−88 (−182 to 6)	−80 (−182 to 21)	NA	−86 (−177 to 5)	29 (−103 to 160)
Pakistani	NA	265 (89 to 441)	269 (78 to 460)	NA	191 (100 to 281)	143 (−103 to 390)
Multiracial	NA	−10 (−96 to 76)	−19 (−104 to 65)	NA	6 (−90 to 101)	158 (67 to 248)
Other or unknown^f^	NA	−891 (−929 to −853)	−899 (−933 to −865)	NA	−785 (−828 to −743)	5 (−90 to 101)
White	[Reference]	[Reference]	[Reference]	[Reference]	[Reference]	[Reference]
Combined log pseudolikelihood	−2 043 932.600	−2 028 190.900	−2 026 676.000	−2 185 315.900	−2 174 796.600	−2 173 081.800
Observations (part 1)^g^, No.	268 231	268 231	268 212	268 231	268 231	267 435
Akaike information criterion	16.646	16.580	16.571	16.529	16.460	16.451
Bayesian information criterion	−2 329 398.000	−2 344 665.000	−2 345 113.000	−2 708 404.000	−2 725 991.000	−2 726 810.000
Deviance (part 2)	550 874.110	535 273.385	532 971.642	532 721.500	514 797.676	512 107.837

Abbreviations: BMI, body mass index (calculated as weight in kilograms divided by height in meters squared); NA, not applicable.

^a^ Data from Clinical Practice Research Datalink–Hospital Episode Statistics Electronic Medical Records, 2014 to 2020.

^b^ To convert to 2022 US dollars, multiply by 1.44.

^c^ Mean marginal difference in costs obtained from unadjusted model.

^d^ Mean marginal difference in costs obtained from adjusting for the main effects of demographic characteristics (sex, age group, ethnicity, the English Index of Multiple Deprivation score, and geographic region).

^e^ Mean marginal difference in costs obtained from adjusting for the main effects of demographic characteristics (sex, age group, ethnicity, the English Index of Multiple Deprivation score, and geographic region) and interactions (3-way interaction of BMI category with sex and age group and lower order interactions; BMI category × sex, BMI category × age group, sex × age group, BMI category × ethnicity, ethnicity × Index of Multiple Deprivation, and Index of Multiple Deprivation × region).

^f^ The other ethnicity category represents children coded as “any other ethnic group” in the Clinical Practice Research Datalink dataset.

^g^ Observations (zero and nonzero costs) used in the logit model. This is usually the entire population of 268 231 unique children but is sometimes slightly less for model 3 when observations are omitted due to collinearity. Observations in part 2 (the generalized linear model) are based solely on observations with nonzero costs and are the same across all 3 models (primary care, year before index BMI record: 216 121 [81%]; primary care, year after index BMI record: 249 405 [93%]; secondary care, year before index BMI record: 152 772 [57%]; secondary care, year after index BMI record: 162 774 [61%]; total health care, year before index BMI record: 233 055 [87%]; and total health care, year after index BMI record: 259 955 [97%]).

**Table 3.** Mean Marginal Difference in Total Health Care in the Year Before Index BMI Measurement (Model 3) by Sex, Age Group, and Ethnicity Among Children Aged 2 to 15 years^a^

Covariate	Cost (95% CI), £^b^				
	>2nd and <85th Centile	≤2nd Centile (underweight)	≥85th and <95th Centile (overweight)	≥95th and <99.6th Centile (obesity)	≥99.6th Centile (severe obesity)
Sex					
Male	[Reference]	71 (−59 to 202)	174 (57 to 291)	171 (88 to 254)	126 (14 to 239)
Female	[Reference]	−97 (−201 to 6)	96 (10 to 182)	41 (−41 to 123)	178 (12 to 344)
Age group, y					
2-3	[Reference]	29 (−242 to 300)	90 (−69 to 249)	156 (−62 to 373)	−20 (−274 to 235)
4-5	[Reference]	−11 (−170 to 147)	236 (111 to 361)	210 (94 to 326)	347 (130 to 564)
6-7	[Reference]	−94 (−214 to 27)	124 (13 to 236)	117 (6 to 228)	238 (−24 to 501)
8-9	[Reference]	−24 (−179 to 131)	130 (−61 to 320)	20 (−54 to 95)	41 (−63 to 145)
10-11	[Reference]	70 (−166 to 307)	83 (−57 to 224)	−7 (−119 to 104)	43 (−90 to 176)
12-13	[Reference]	223 (−338 to 784)	78 (−315 to 471)	322 (−47 to 691)	201 (−139 to 541)
14-15	[Reference]	4861 (−3541 to 13264)	52 (−554 to 658)	849 (−25 to 1723)	93 (−590 to 776)
Ethnicity					
Bangladeshi	[Reference]	143 (−300 to 587)	545 (−361 to 1452)	26 (−256 to 308)	−189 (−421 to 43)
Black	[Reference]	−100 (−351 to 152)	−102 (−284 to 81)	−20 (−208 to 168)	−27 (−246 to 193)
Chinese or other Asian	[Reference]	−145 (−411 to 122)	431 (−65 to 927)	231 (−102 to 564)	143 (−309 to 595)
Indian	[Reference]	−106 (−359 to 147)	−295 (−492 to −97)	329 (−34 to 691)	240 (−291 to 772)
Pakistani	[Reference]	−224 (−447 to 0)	1155 (−512 to 2821)	173 (−103 to 450)	−14 (−343 to 315)
White	[Reference]	54 (−80 to 187)	104 (40 to 169)	133 (57 to 208)	176 (65 to 287)
Multiracial	[Reference]	−253 (−610 to 104)	173 (−87 to 434)	45 (−251 to 340)	228 (−31 to 487)
Other or unknown^c^	[Reference]	−50 (−133 to 34)	82 (−36 to 200)	33 (−36 to 102)	156 (−56 to 369)

Abbreviation: BMI, body mass index (calculated as weight in kilograms divided by height in meters squared).

^a^ Data from Clinical Practice Research Datalink–Hospital Episode Statistics Electronic Medical Records, 2014 to 2020.

^b^ To convert to 2022 US dollars, multiply by 1.44.

^c^ The other ethnicity category represents children coded as “any other ethnic group” in the Clinical Practice Research Datalink dataset.

**Table 4.** Mean Marginal Difference in Total Health Care Costs in the Year After Index BMI Measurement (Model 3) by Sex, Age Group, and Ethnicity Among Children Aged 2 to 15 Years^a^

Covariate	Costs (95% CI), £^b^				
	2nd and <85th Centile	≤2nd Centile (underweight)	≥85th and <95th Centile (overweight)	≥95th and <99.6th Centile (obesity)	≥99.6th Centile (severe obesity)
Sex					
Male	[Reference]	144 (25 to 264)	54 (−11 to 120)	142 (52 to 232)	138 (5 to 272)
Female	[Reference]	189 (52 to 326)	83 (7 to 158)	139 (36 to 243)	253 (44 to 462)
Age group, y					
2-3	[Reference]	277 (28 to 525)	18 (−101 to 137)	122 (−42 to 287)	13 (−170 to 197)
4-5	[Reference]	191 (26 to 356)	163 (45 to 280)	334 (140 to 528)	472 (171 to 772)
6-7	[Reference]	91 (−54 to 236)	122 (15 to 230)	146 (22 to 270)	288 (−73 to 649)
8-9	[Reference]	145 (−46 to 337)	−7 (−93 to 80)	50 (−52 to 152)	21 (−69 to 111)
10-11	[Reference]	105 (−149 to 360)	11 (−111 to 134)	−34 (−129 to 61)	47 (−80 to 173)
12-13	[Reference]	308 (−403 to 1019)	−256 (−555 to 43)	57 (−253 to 367)	−1 (−412 to 411)
14-15	[Reference]	952 (−629 to 2532)	72 (−526 to 671)	−138 (−541 to 265)	−216 (−676 to 245)
Ethnicity					
Bangladeshi	[Reference]	−122 (−435 to 191)	118 (−347 to 582)	28 (−331 to 386)	−261 (−537 to 16)
Black	[Reference]	269 (−144 to 681)	1 (−174 to 177)	−3 (−174 to 168)	208 (−76 to 492)
Chinese or other Asian	[Reference]	123 (−241 to 486)	428 (−69 to 926)	798 (−320 to 1916)	139 (−275 to 552)
Indian	[Reference]	−8 (−304 to 287)	−88 (−305 to 129)	135 (−159 to 428)	55 (−239 to 348)
Pakistani	[Reference]	−51 (−311 to 208)	85 (−212 to 381)	209 (−97 to 516)	115 (−243 to 474)
Multiracial	[Reference]	22 (−456 to 501)	−152 (−334 to 29)	−251 (−430 to −73)	90 (−163 to 344)
White	[Reference]	217 (82 to 352)	85 (21 to 148)	170 (88 to 253)	221 (95 to 348)
Other or unknown^c^	[Reference]	69 (−37 to 174)	28 (−51 to 107)	50 (−27 to 126)	177 (−72 to 426)

Abbreviation: BMI, body mass index (calculated as weight in kilograms divided by height in meters squared).

^a^ Data from Clinical Practice Research Datalink–Hospital Episode Statistics Electronic Medical Records, 2014 to 2020.

^b^ To convert to 2022 US dollars, multiply by 1.44.

^c^ The other ethnicity category represents children coded as “any other ethnic group” in the Clinical Practice Research Datalink dataset.

Relative to healthy weight, higher health care costs were observed in children in the overweight, obesity, and severe obesity categories both before and after BMI measurement (Table 2). In the year before BMI measurement, statistically significant excess costs were estimated at £139 (95% CI, £53-£225) (US $200 [95% CI, US $76-US $324]) for overweight, £113 (95% CI, £54-£172) (95% CI, US $163 [95% CI, US $78-US $248]) for obesity, and £150 (95% CI, £56-£243) (95% CI, US $216 [95% CI, US $81-US $350]) for severe obesity. In the year after BMI measurement, excess costs were £67 (95% CI, £17-£116) (US $97 [95% CI, US $24-US $167]) for overweight, £141 (95% CI, £68-£213) (US $203 [95% CI, US $98-US $307]) for obesity, and £190 (95% CI, £77-£302) (US $274 [95% CI, US $111-US $435]) for severe obesity. Children with underweight showed significantly higher costs only in the year after BMI measurement (£164 [95% CI, £69-£260]) (US $236 [US $99-US $374]). Overall, girls incurred lower costs than boys, with cost differences of £79 (95% CI, £47-£112) (US $114 [US $68-US $161]) and £76 (95% CI, £42-£109) (US $110 [95% CI, $60-US $157]) before and after BMI measurement, respectively.

We also explored how sex, age, and ethnicity modified these associations. Before BMI measurement, boys and girls with overweight had higher costs (£174 [95% CI, £57-£291]) (US $251 [95% CI, US $82-US $419]) and £96 (95% CI, £10-£182) (US $138 [95% CI, US $14-US $262], respectively) compared with their healthy-weight peers, with boys incurring greater excess costs (Table 3). Among children with obesity, statistically significant excess costs were observed only in boys (£171 [95% CI, £88–£254]) (US $246 [95% CI, US $127–US $366]), while severe obesity was associated with higher statistically significant costs for both sexes, with girls showing a greater excess (£178 [95% CI, £12-£344]) (US $256 [95% CI, US $17-US $495]). After BMI measurement (Table 4), boys (£144 [95% CI, £25-£264]) (US $207 [95% CI, US $36-US $380]) and girls (£189 [95% CI, £52-£326]) (US $272 [US $75-US $469]) with underweight exhibited significantly higher costs. Excess costs related to overweight were significant only for girls (£83 [95% CI, £7-£158]) (US $120 [95% CI, US $10-US $228]). Obesity and severe obesity were associated with statistically significant excess costs for both sexes, with the highest excess observed in girls with severe obesity (£253 [95% CI, £44-£462]) (US $364 [95% CI, US $63-US $665]).

Before BMI measurement (Table 3), children aged 4 to 5 years with overweight or obesity incurred statistically significantly higher costs than their healthy-weight counterparts, with the greatest excess seen in children with severe obesity (£347 [95% CI, £130-£564]) (US $500 [95% CI, US $187-US $812]). After BMI measurement (Table 4), statistically significant higher costs were observed in children with underweight aged 2 to 3 years and 4 to 5 years as well as in children with overweight or obesity aged 4 to 5 years and 6 to 7 years. Severe obesity was associated with significantly higher costs exclusively in children aged 4 to 5 years (£472 [95% CI, £171-£772) (US $680 [95% CI, US $246-US $1111]).

In relation to ethnicity, before BMI measurement (Table 3), White children exhibited significantly higher costs in the overweight (£104 [95% CI, £40-£169]) (US $150 [95% CI, US $58-US $244]), obesity (£133 [95% CI, £57-£208]) (US $191 [US $82-US $300]), and severe obesity (£176 [95% CI, £65-£287]) ($253 [95% CI, US $94-US $414]) categories compared with healthy-weight peers. Conversely, Indian children living with overweight had significantly lower costs (£295 [95% CI, £97-£492]) (US $425 [US $140-US $709]) than their healthy-weight peers. After BMI measurement (Table 4), White children continued to show elevated costs across most BMI categories, whereas children of mixed ethnicity exhibited lower costs in the obesity category (£251 [95% CI, £73-£430]) (US $361 [95% CI, US $105-US $619]). Significant differences were not observed for other ethnic groups.

The total annual national health care costs attributable to childhood excess weight in England was £0.273 billion (95% CI, £0.121-£0.426 billion) (US $0.393 billion [95% CI, $0.174 billion to $0.614 billion]) post-BMI measurement (Figure; eTables 13-14 in Supplement 1). The higher costs in the year before were accounted for by substantially higher excess costs due to overweight.

**Figure.**
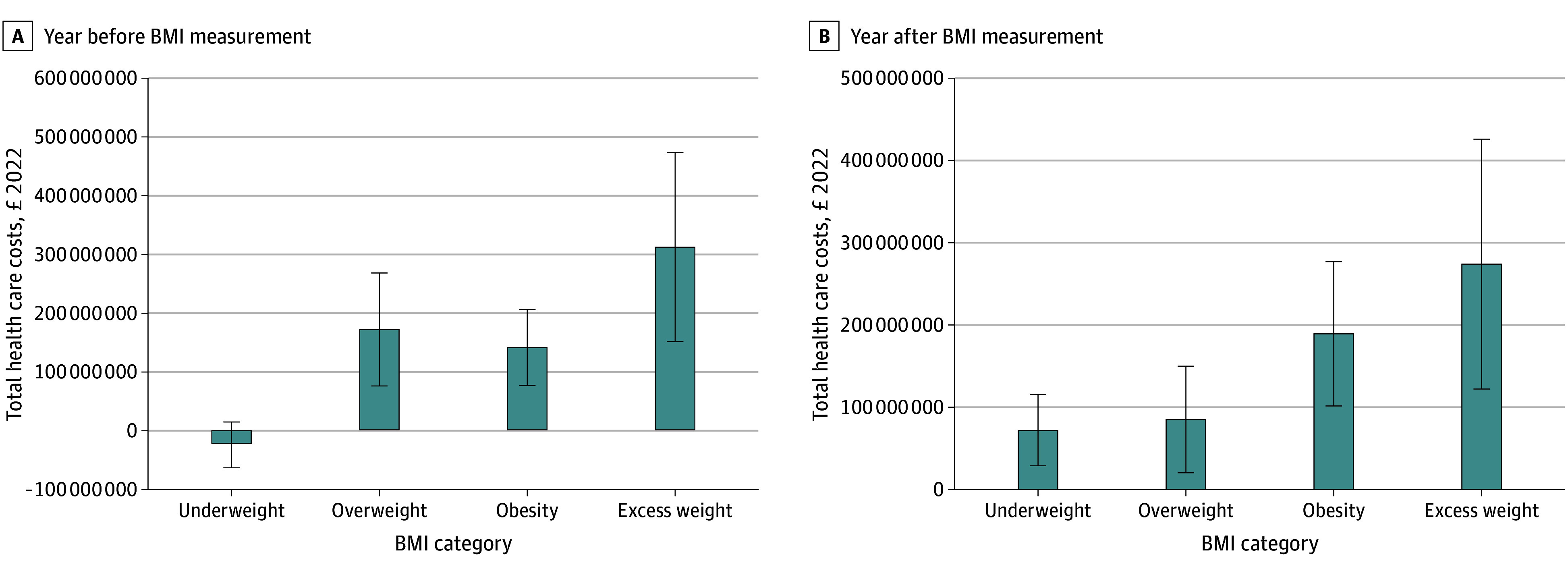
Marginal Differences in Total Health Care Costs by Childhood Weight Status, England, 2014-2020 Estimates from model 3 were adjusted for demographic characteristics and their interactions using Clinical Practice Research Datalink–Hospital Episodes Statistics linked records. Marginal differences represent excess costs when children in the healthy-weight category are assumed to have £0 in primary and secondary health care use costs. Errors bars indicate 95% CIs. To convert to 2022 US dollars, multiply by 1.44.

## Discussion

This study is the first, to our knowledge, to estimate national health care costs associated with childhood weight status using English electronic health records containing measured BMI. Unlike previous studies,^14,15,16,18,20^ our analysis included a temporal assessment of annual costs relative to BMI measurement, revealing patterns before and after assessment. Premeasurement costs establish a baseline for routine health care use, reflecting the burden of disease before BMI classification. Postmeasurement costs capture the impact of BMI assessment, including additional care, and are crucial for evaluating the cost-effectiveness of BMI-based interventions. We found higher health care costs in the year after BMI measurement compared with the preceding year, particularly for children classified as having underweight or obesity. Excess costs varied by sex, age group, and ethnicity in both periods. Covariate interactions were apparent, albeit limited to some covariate levels and BMI categories.

These findings align with and build on previous research.^16^ The Born in Bradford study by Hasan,^16^ a cohort of 9397 children born in the fifth most income-deprived district in England, also reported higher costs associated with underweight and obesity. However, the restricted age range (4-8 years) and smaller sample size limit direct comparisons. Differences in cost structures and weight distributions between health care systems in the UK and other countries further complicate cross-study comparisons.

Our findings of excess health care costs for children living with underweight compared with those with healthy-weight post-BMI measurement echo results from Hasan^16^ and researchers in the US. For example, Kumar et al^20^ reported excess costs, with the highest costs observed for severe obesity followed by underweight. Another US-based study^18^ reported a similar order of magnitude for excess costs, but the estimates for underweight were not statistically significant. Likewise, a systematic review by Kent et al^19^ found that adults with underweight incurred 13% higher annual health care costs than those with healthy weight, consistent with our estimate of an approximate 10% excess cost for children living with underweight. However, caution is warranted when comparing these findings, because the authors did not use a pre- and post-BMI measurement approach and health care use patterns may differ between children and adults.

Our findings of relative excess costs associated with overweight and obesity also align with recent US-based studies, underscoring the economic burden of childhood obesity.^18,39^ Biener et al^39^ estimated annual medical costs for obesity and severe obesity at £766 (US $935) and £1259 (US $1536) (2022 dollars) for children aged 11 to 17 years, including third-party and out-of-pocket expenditures. These estimates highlight the substantial externalities of childhood obesity, supporting the case for governmental intervention.^54^ Ward and colleagues^18^ similarly estimated health care costs associated with excess weight in children aged 6 to 19 years, reporting excess costs of £91.32 per child. The higher estimates reported by Biener et al^39^ may reflect methodological differences, including their use of instrumental variable approaches, which typically yield higher estimates.^37^

Our analyses revealed that sex, age, and ethnicity significantly modified the association between weight status and health care costs, although interactions were limited to specific covariate levels. These variations may reflect biological, behavioral, or health care access differences, highlighting the need for targeted rather than one-size-fits-all interventions. Consistent with the study by Kumar et al,^20^ girls incurred higher costs for severe obesity but lower costs for underweight compared with boys after BMI measurement. Although Hasan^16^ found no variation due to ethnicity, his model excluded interaction terms, unlike ours. In our study, statistical significance was not consistently observed across all covariate levels. For instance, excess costs were estimated in BMI categories for only White and Indian children. These findings suggest that although standardized cost estimates may be appropriate in economic evaluations of interventions addressing childhood excess weight, subgroup-specific estimates, such as those presented here, may be especially valuable for tailored strategies.

The economic burden of childhood excess weight is considerable. In England, NHS costs attributed to elevated BMI, including children and adults, were estimated at £6.1 billion in 2015 and are projected to increase to £9.7 billion by 2050.^55,56,57^ By applying our cost weights to the 2.5 million children in England affected by excess weight, we estimated that excess national health care costs could reach £0.3 billion annually. Although this figure is modest compared with estimates from the US,^18,20,39^ it represents a substantial opportunity cost on the NHS budget. Preventive measures targeting childhood excess weight could also yield significant long-term savings by reducing adult obesity rates and associated health care costs.^19^

### Implications of Research

Our findings have practical implications for health care planning and resource allocation, such as the delineation of pre- and post-BMI measurement costs suited for capturing baseline health care use and the impact of BMI assessment, respectively. Furthermore, by providing tailored cost assessments for childhood weight status, our study offers valuable inputs for cost-effectiveness analyses of interventions targeting childhood unhealthy weight. These estimates can also guide resource allocation strategies within specific population subgroups.

### Limitations

Our study has limitations. Despite the overall large sample, the number of children aged 14 to 15 years was small. BMI is not routinely recorded in primary care in England, which may introduce selection bias. Furthermore, BMI is more likely to be measured during illness or among children at weight extremes.^53^ Nonetheless, previous studies^25,53,58^ have shown BMI distributions in the CPRD to be comparable to other UK datasets, such as the Health Survey for England.

Not all children with excess weight, particularly milder cases, seek care for weight-related issues. Parental attitudes and other barriers may limit health care contact,^59,60^ potentially leading to underrepresentation in our sample. Future research should explore these factors to better understand variations in health care use among children with excess weight. Longitudinal studies would also help assess lagged and cumulative cost patterns throughout a lifetime.

## Conclusions

Using nationwide primary care records linked with secondary care data, this study found robust evidence on the economic burden of childhood weight status in England. Health care costs were higher for all BMI categories, including underweight, compared with healthy weight in the year after BMI measurement. Extrapolated to the national childhood population, excess weight accounts for an estimated £0.3 billion in annual health care costs. These findings underscore the economic rationale for preventive and curative interventions targeting childhood unhealthy weight, with cost estimates serving as valuable inputs for cost-effectiveness analyses.

## Notes

Supplement 1. **eFigure 1.** Time series prevalence of weight status categories in Reception from 2006/07 to 2022/23 **eFigure 2.** Time series prevalence of weight status categories in Year 6 from 2006/07 to 2022/23 **eFigure 3.** Logic model of dataset selection **eTable 1.** BMI distributions for the CPRD-HES study population compared with the NCMP 2022/23 survey prevalence for children at ages 4-5 and 10-11 **eTable 2.** Logic Model detailing Primary Care Consultations Inclusion, Exclusion, and Costing Process **eTable 3.** Logic Model detailing Primary Care Prescriptions Inclusion, Exclusion, and Costing Process **eTable 4.** Logic Model detailing Primary Care Tests and Investigations Inclusion, Exclusion, and Costing Process **eTable 5.** Logic Model for Secondary Care Admitted Patient Care Inclusion, Exclusion, and Costing Process **eTable 6.** Logic Model for Secondary Care Outpatient Care Inclusion, Exclusion, and Costing Process **eTable 7.** Logic Model for Secondary Care Accident and Emergency Care Inclusion, Exclusion, and Costing Process **eTable 8.** Box-Cox test on model 3 for testing the suitability of the link function **eTable 9.** Modified Park Test to test for the gamma distribution **eTable 10.** AIC and BIC comparisons for models 1 to 3 – year before BMI measurement **eTable 11.** AIC and BIC comparisons for models 1 to 3 – year after BMI measurement **eTable 12.** Predicted absolute annual total health care costs by BMI category, in the year before and after BMI measurement, from Model 3 (preferred covariate- and interaction-adjusted model) **eTable 13.** Marginal Difference in Predicted Total Healthcare Costs (GBP) Attributable to Underweight and Excess Weight Categories in the year before BMI measurement (Model 3), Clinical Practice Research Datalink (CPRD)-Hospital Episode Statistics (HES) electronic medical records (2014-2020) **eTable 14.** Marginal Difference in Predicted Total Healthcare Costs (GBP) Attributable to Underweight and Excess Weight Categories in the year after BMI measurement (Model 3), Clinical Practice Research Datalink (CPRD)-Hospital Episode Statistics (HES) electronic medical records (2014-2020) **eResults.** Assessment of sample representativeness
eReference

Supplement 2.
Data Sharing Statement
