# Supplementary material for: Child Body Mass Index and Health Care Costs in England
Source: JAMA Netw Open. 2025 Oct 14;8(10):e2537560. doi: 10.1001/jamanetworkopen.2025.37560 (PMC12522009; doi:10.1001/jamanetworkopen.2025.37560)
Supplement: Supplement 1. — eFigure 1. Time series prevalence of weight status categories in Reception from 2006/07 to 2022/23 eFigure 2. Time series prevalence of weight status categories in Year 6 from 2006/07 to 2022/23 eFigure 3. Logic model of dataset selection eTable 1. BMI distributions for the CPRD-HES study population compared with the NCMP 2022/23 survey prevalence for children at ages 4-5 and 10-11 eTable 2. Logic Model detailing Primary Care Consultations Inclusion, Exclusion, and Costing Process eTable 3. Logic Model detailing Primary Care Prescriptions Inclusion, Exclusion, and Costing Process eTable 4. Logic Model detailing Primary Care Tests and Investigations Inclusion, Exclusion, and Costing Process eTable 5. Logic Model for Secondary Care Admitted Patient Care Inclusion, Exclusion, and Costing Process eTable 6. Logic Model for Secondary Care Outpatient Care Inclusion, Exclusion, and Costing Process eTable 7. Logic Model for Secondary Care Accident and Emergency Care Inclusion, Exclusion, and Costing Process eTable 8. Box-Cox test on model 3 for testing the suitability of the link function eTable 9. Modified Park Test to test for the gamma distribution eTable 10. AIC and BIC comparisons for models 1 to 3 – year before BMI measurement eTable 11. AIC and BIC comparisons for models 1 to 3 – year after BMI measurement eTable 12. Predicted absolute annual total health care costs by BMI category, in the year before and after BMI measurement, from Model 3 (preferred covariate- and interaction-adjusted model) eTable 13. Marginal Difference in Predicted Total Healthcare Costs (GBP) Attributable to Underweight and Excess Weight Categories in the year before BMI measurement (Model 3), Clinical Practice Research Datalink (CPRD)-Hospital Episode Statistics (HES) electronic medical records (2014-2020) eTable 14. Marginal Difference in Predicted Total Healthcare Costs (GBP) Attributable to Underweight and Excess Weight Categories in the year after BMI measurement (Model 3), Clinical Practic [file jamanetwopen-e2537560-s001.pdf]

Supplemental Online Content

Onyimadu O, Hayes A, Fahr P, et al. Child body mass index and health care costs in England, 2014-2020. *JAMA Netw Open*. 2025;8(10):e2537560. doi:10.1001/jamanetworkopen.2025.37560

eFigure 1. Time series prevalence of weight status categories in Reception from 2006/07 to 2022/23
eFigure 2. Time series prevalence of weight status categories in Year 6 from 2006/07 to 2022/23
eFigure 3. Logic model of dataset selection
eTable 1. BMI distributions for the CPRD-HES study population compared with the NCMP 2022/23 survey prevalence for children at ages 4-5 and 10-11
eTable 2. Logic Model detailing Primary Care Consultations Inclusion, Exclusion, and Costing Process
eTable 3. Logic Model detailing Primary Care Prescriptions Inclusion, Exclusion, and Costing Process
eTable 4. Logic Model detailing Primary Care Tests and Investigations Inclusion, Exclusion, and Costing Process
eTable 5. Logic Model for Secondary Care Admitted Patient Care Inclusion, Exclusion, and Costing Process
eTable 6. Logic Model for Secondary Care Outpatient Care Inclusion, Exclusion, and Costing Process
eTable 7. Logic Model for Secondary Care Accident and Emergency Care Inclusion, Exclusion, and Costing Process
eTable 8. Box-Cox test on model 3 for testing the suitability of the link function
eTable 9. Modified Park Test to test for the gamma distribution
eTable 10. AIC and BIC comparisons for models 1 to 3 – year before BMI measurement
eTable 11. AIC and BIC comparisons for models 1 to 3 – year after BMI measurement
eTable 12. Predicted absolute annual total health care costs by BMI category, in the year before and after BMI measurement, from Model 3 (preferred covariate- and interaction-adjusted model).
eTable 13. Marginal Difference in Predicted Total Healthcare Costs (GBP) Attributable to Underweight and Excess Weight Categories in the year before BMI measurement (Model 3), Clinical Practice Research Datalink (CPRD)-Hospital Episode Statistics (HES) electronic medical records (2014-2020)
eTable 14. Marginal Difference in Predicted Total Healthcare Costs (GBP) Attributable to Underweight and Excess Weight Categories in the year after BMI measurement (Model 3), Clinical Practice Research Datalink (CPRD)-Hospital Episode Statistics (HES) electronic medical records (2014-2020)
eResults. Assessment of sample representativeness
eReference

This supplemental material has been provided by the authors to give readers additional information about their work.

© 2025 Onyimadu O et al. *JAMA Network Open*.

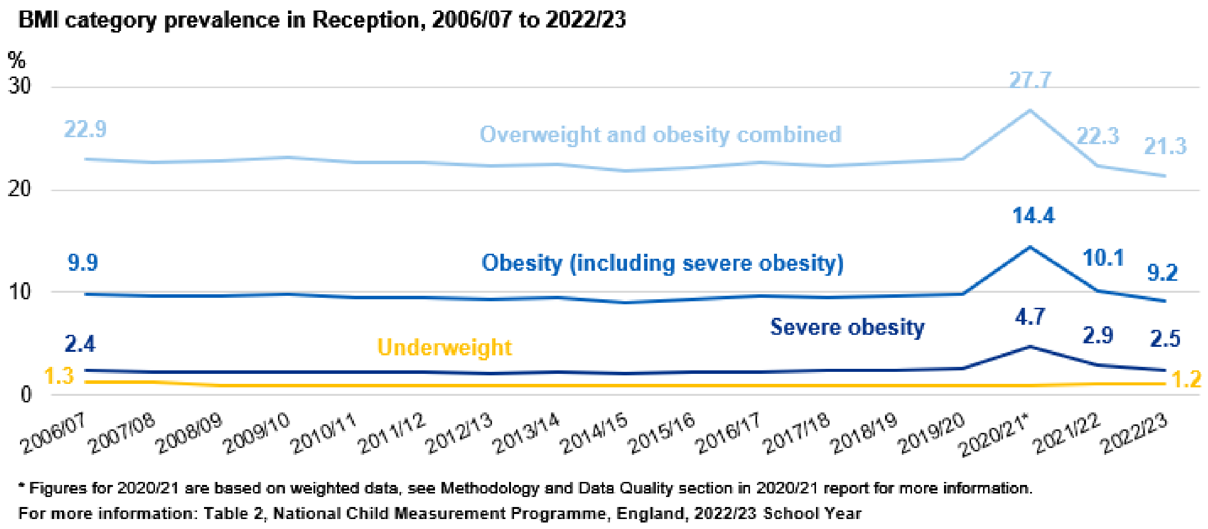

eFigure 1: *Time series prevalence of weight status categories in Reception from 2006/07 to 2022/23 (source: [1])*

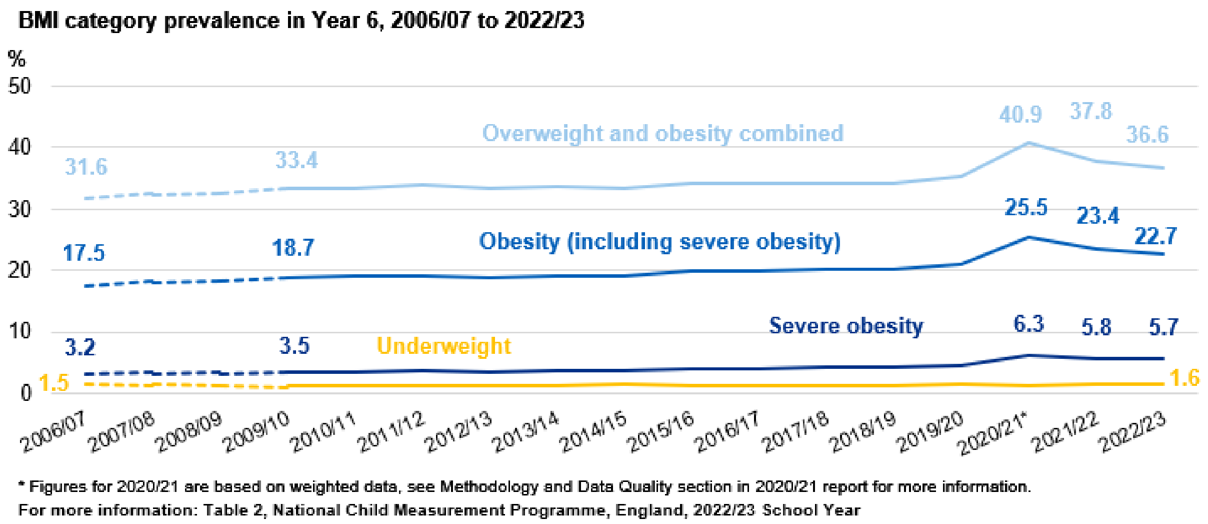

eFigure 2: *Time series prevalence of weight status categories in Year 6 from 2006/07 to 2022/23 (source: [1])*

© 2025 Onyimadu O et al. *JAMA Network Open*.

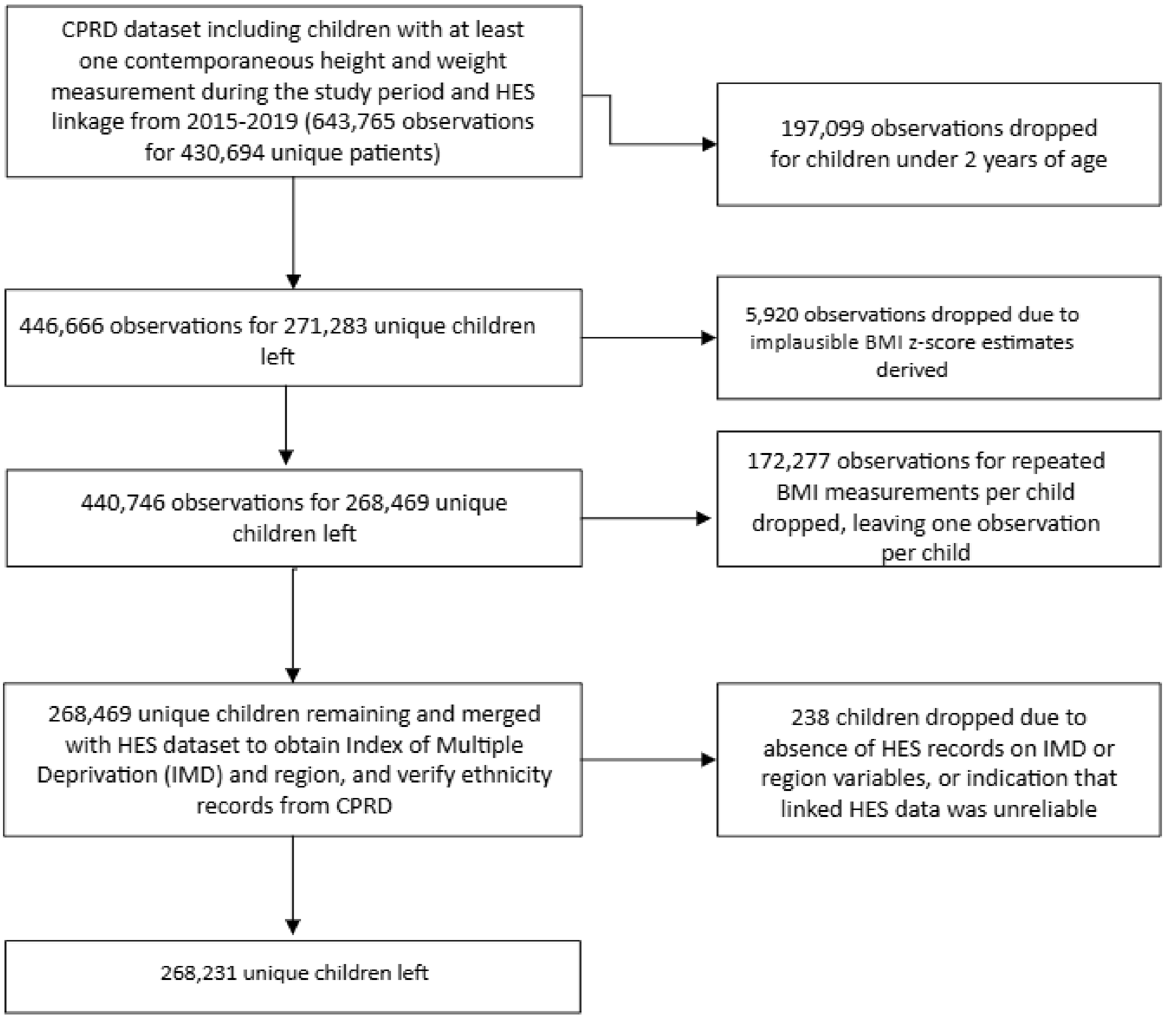

eFigure 3: *Logic model of dataset selection*

© 2025 Onyimadu O et al. *JAMA Network Open*.

eTable 1: *BMI distributions for the CPRD-HES study population compared with the NCMP 2022/23 survey prevalence for children at ages 4-5 and 10-11*

Variable	Variable levels	n	%	NCMP 2022/23 survey (%)
**Distribution of BMI category at ages 4-5 (reception) - boys and girls**				
	Underweight	2,997	4.85	1.20
	Healthy weight	44,258	71.60	77.50
	Overweight	6,833	11.05	12.20
	Obesity	7,726	12.50	9.20
**Distribution of BMI category at ages 10-11 (year 6) - boys and girls**				
	Underweight	1,213	3.60	1.60
	Healthy weight	19,488	57.82	61.90
	Overweight	4,352	12.91	13.90
	Obesity	8,650	25.67	22.70

© 2025 Onyimadu O et al. *JAMA Network Open*.

eTable 2: *Logic Model detailing Primary Care Consultations Inclusion, Exclusion, and Costing Process*

Sequence	Component	Dataset/variables of interest in dataset	Activity	Output	Outcome
1	**Definition of Duplicate Consultations**	Patient ID (patid), Consultation ID (consid), Consultation date (consdate), Staff ID (staffid), Staff job category (jobcatid), EMIS medical dictionary term.	Identify duplicate consultations based on variables and exclude duplicates.	Dataset with duplicate consultations excluded.	Improved accuracy by removing redundant records.
2	**First Decision Rule: Administrative Activities**	EMIS medical dictionary terms.	Work with clinicians to review activity terms in the consultation dataset and identify administrative or overhead tasks that are not directly patient-facing. Exclude these activities from the analysis.	Dataset with only patient-facing consultation records.	Focus on consultations relevant to patient care.
3	**Second Decision Rule: Job Categories**	Staff job category (jobcatid).	Exclude records with missing staff/job category information. Exclude consultations conducted by support staff (e.g., receptionists, clerical workers).	Dataset with only clinical consultations by healthcare professionals.	Focus on clinically relevant consultations.
4	**Categorization of Consultation Types**	Consultation type based on EMIS medical dictionary terms (surgical, home, phone).	Assign consultations to categories: Surgical, Home, and Phone.	Dataset with consultations classified by type.	Differentiated consultations for cost analysis based on type.
5	**Costing of Consultations**	Unit Costs of Health and Social Care (PSSRU 2021). Staff bands/grades data from NHS websites and previous studies.	Assign costs to consultation types: Surgical, home, and phone consultations based on duration, setting, and associated costs. Use average cost if consultation type is uncertain or missing.	Dataset with consultation costs assigned and adjusted to 2021/22 prices using NHS Cost Inflation Index.	Estimation of costs for each consultation type with improved accuracy.
6	**Timeframe Categorization**	Consultation date (consdate or obsdate).	Categorise consultations into periods occurring **before** or **after** a BMI measurement.	Dataset with consultations grouped by timeframe.	Enables analysis of healthcare utilization relative to BMI measurement.

© 2025 Onyimadu O et al. *JAMA Network Open*.

eTable 3: *Logic Model detailing Primary Care Prescriptions Inclusion, Exclusion, and Costing Process*

Sequence	Component	Dataset/variables of interest in dataset	Activity	Output	Outcome
**1**	**Data Cleaning and Deduplication**	Patient data (Patient identifier (variable 'patid'), the date associated with the prescription ('issuedate'), the CPRD code to describe the treatment ('prodcodeid'), the EMIS product dictionary term (description of the observation, event or activity), the total quantity entered by the GP for the prescribed treatment ('quantity') and the estimated cost of the treatment to the NHS ('estnhscost')) from CPRD	Dropped duplicate prescription observations based on identical variables: patid, issuedate, prodcodeid, quantity, estnhscost, and others.	Cleaned dataset of unique prescription entries.	Improved data accuracy and reliability for analysis.
**2**	**Exclusion Criteria**	Prescription cost (estnhscost) and quantity (quantity) data	Excluded observations where quantity was -1 and set quantity to zero if estnhscost was zero.	Dataset with invalid or ambiguous prescription data excluded.	Refined dataset ready for cost analysis of prescriptions.
**3**	**Timeframe Categorization**	Prescription issue date (issuedate) and BMI measurement date	Categorized prescriptions based on whether they occurred in the year before or after a BMI measurement (issuedate).	Categorized dataset distinguishing pre- and post-BMI measurement prescriptions.	Enables temporal analysis of prescription trends relative to BMI measurement.
**4**	**Cost Calculation**	CPRD-provided prescription costs (estnhscost)	Used CPRD-provided cost data to estimate NHS prescription costs for the study period.	Dataset with NHS prescription cost estimates integrated.	Facilitates understanding of the economic burden of prescriptions in the context of childhood BMI.

© 2025 Onyimadu O et al. *JAMA Network Open*.

eTable 4: *Logic Model detailing Primary Care Tests and Investigations Inclusion, Exclusion, and Costing Process*

Sequence	Component	Dataset/variables of interest in dataset	Activity	Output	Outcome
**1**	**Data Cleaning and Deduplication**	Data from CPRD (test requests, observations) and EMIS product dictionary	Exclusion of duplicates by patient ID (patid), test/investigation date (obsdate), and EMIS product dictionary terms.	Cleaned and categorized dataset of primary care tests/investigations.	Improved data accuracy and reliability for analysis.
**2**	**Costing Tests and Investigations**	NHS Reference Cost 2020/21, NHS Cost Inflation Index 2021/22, CPRD code list, EMIS product dictionary	Derivation of unit costs and categorization of tests. Extraction of direct access unit costs and matching with medcodeid from CPRD. Averaging costs where multiple unit costs exist for the same test.	Mapped unit costs for tests and investigations, with averages applied where specific codes were missing or ambiguous. Adjusted costs to 2021/22 prices where necessary.	Accurate estimation of the economic burden of primary care tests and investigations related to childhood BMI assessment.
**3**	**Timeframe Categorization**	CPRD data on test dates (obsdate)	Categorization of tests and investigations as occurring in the year before or after BMI measurement, based on dates in the dataset.	Segregated data indicating timing of tests in relation to BMI measurement (pre- or post-BMI test year).	Enhanced ability to analyze temporal patterns and healthcare costs linked to childhood BMI measurements.

© 2025 Onyimadu O et al. *JAMA Network Open*.

eTable 5: *Logic Model for Secondary Care Admitted Patient Care Inclusion, Exclusion, and Costing Process*

Sequence	Component	Dataset/variables of interest in dataset	Activity	Output	Outcome
**1**	**HRG Code Generation**	HRG4+ 2021/22 Reference Cost Grouper; FCE data; HES admitted patient care variables (episode key uniquely identifying an episode of care ('epikey'), episode start date ('epistart') and end date ('epiend')); Grouper User Manual	Generate HRG codes at the FCE level using the HRG4+ Grouper; ensure no duplicates in dataset by checking 'epikey' and 'patid'; include only episodes with valid 'epistart' and 'epiend'.	HRG codes derived for all valid episodes in the dataset.	Accurate categorization of admitted patient episodes for subsequent cost analysis.
**2**	**HRG to Cost Linkage**	HRG codes; National average unit costs (NAUCs); Department descriptions (e.g., elective/non-elective classifications); HES data variables	Link HRG codes to NAUCs by determining department descriptions; exclude childbirth-related admissions.	HRG codes linked to appropriate department descriptions and unit costs.	Accurate allocation of national average unit costs to HRG-based hospital admissions.
**3**	**Cost Adjustment**	NHS Cost Inflation Index (NHSCII); 2021/22 National Cost Collection Data	Adjust unit costs (NAUCs) to 2021/22 prices using the NHSCII.	Unit costs adjusted to 2021/22 prices for all hospital admissions.	Cost data standardization for meaningful comparison across episodes and periods.
**4**	**Timeframe Categorization**	Admission episode dates ('epistart' and 'epiend'); BMI measurement date	Categorize hospital admissions as occurring either during the year before or the year after a BMI measurement based on episode start and end dates.	Admissions categorized by their temporal relationship to BMI measurement.	Insight into the timing of hospital care relative to BMI assessment.

HRG4+: Healthcare Resource Groups version 4+; FCE: Finished consultation episode

© 2025 Onyimadu O et al. *JAMA Network Open*.

eTable 6: *Logic Model for Secondary Care Outpatient Care Inclusion, Exclusion, and Costing Process*

Sequence	Component	Dataset/variables of interest in dataset	Activity	Output	Outcome
**1**	**Derivation of HRG Codes**	Outpatient variables from CPRD and the Grouper User Manual.	Use Grouper software to derive HRG codes based on outpatient variables.	Complete list of HRG codes derived from outpatient data.	Enables linkage to unit costs for outpatient care.
**2**	**Linking HRG Codes to Unit Costs**	Patient attendance status ('attended'), medical staff type ('stafftyp'), treatment specialty ('tretspef').	Link HRG codes to outpatient care unit costs (NAUCs) using attendance status, staff type, and treatment speciality. Exclude observations for cancelled or postponed appointments.	Unit costs (NAUCs) linked to outpatient HRG codes based on attendance and staffing variables.	Unit costs for outpatient visits generated.
**3**	**Deriving Costs for Outpatient Procedures**	Treatment specialty and HRG variables.	Match HRG variables for outpatient procedures (OPROC) with appropriate NAUCs based on treatment speciality.	Unit costs (NAUCs) associated with outpatient procedures determined.	Unit costs for outpatient procedures generated.
**4**	**Handling Missing Data**	Missing 'stafftyp', department descriptions, or uncosted service descriptions.	Estimate weighted average costs for HRGs with the same service and currency descriptions. For uncosted services, calculate proxy costs based on total weighted averages of combined outpatient care and procedures.	Proxy unit costs (NAUCs) estimated for missing or uncosted services.	Complete dataset with approximated unit costs for all outpatient services.
**5**	**Timeframe Categorization**	Appointment date ('apptdate').	Categorize outpatient care as occurring in the year before or after BMI measurement based on appointment date.	Outpatient care events categorized by timing relative to BMI measurement.	Enables temporal analysis of outpatient care costs in relation to BMI.

© 2025 Onyimadu O et al. *JAMA Network Open*.

eTable 7: *Logic Model for Secondary Care Accident and Emergency Care Inclusion, Exclusion, and Costing Process*

Sequence	Component	Dataset/variables of interest in dataset	Activity	Output	Outcome
**1**	**Accident and Emergency Care Variables**	Complete list of accident and emergency care variables. Grouper software and Grouper User Manual.	Use the Grouper software to derive HRG codes based on accident and emergency care variables as detailed in the Grouper User Manual.	Comprehensive HRG codes for accident and emergency care.	Consistent classification of accident and emergency care activities.
**2**	**Unit Cost Data**	2021/22 National Cost Collection Data.	Generate Health Resource Group (HRG) non-admitted care codes using HRG4+ 2021/22 Reference Cost Grouper based on accident and emergency department type.	Accurate unit costs for outpatient care based on HRG codes.	Comprehensive estimation of accident and emergency care costs for economic analysis.
**3**	**Handling of Missing Costs**	NHS Reference Cost 2021/22 schedule. Weighted national average costs for all Service codes.	Apply weighted national average costs where specific NAUCs for accident and emergency attendance are unavailable in the NHS Reference Cost schedule.	Filled gaps in cost data for accident and emergency attendances.	Complete dataset with approximated unit costs for all accident and emergency attendances.
**4**	**Timeframe Categorization**	Patient arrival date in accident and emergency department ('arrivaldate').	Categorize accident and emergency care based on whether it occurred in the year before or after a BMI measurement.	Categorized accident and emergency care records for pre- and post-BMI measurement periods.	Temporal analysis of healthcare usage relative to BMI measurement.

© 2025 Onyimadu O et al. *JAMA Network Open*.

eTable 8: *Box-Cox test on model 3 for testing the suitability of the link function*

	Total_costs	Coefficient	Standard error	Lower 95% CI	Upper 95% CI
Year before BMI measurement	Theta	0.0935628	0.000948	0.0917048	0.0954209
Year after BMI measurement	Theta	0.1037986	0.000935	0.1019664	0.1056309

The log link function is suitable in both cases, as theta is sufficiently close to 0. While the power link function is also applicable, it yields poorer results based on AIC and BIC criteria.

eTable 9: *Modified Park Test to test for the gamma distribution*

	raw2	Coefficient	Standard error	Lower 95% CI	Upper 95% CI
Year before BMI measurement	lyhat (β_1)	1.319894	0.435279	0.4667624	2.173026
	_cons	7.36103	3.147242	1.192549	13.52951
Year after BMI measurement	lyhat (β_1)	0.5780173	0.696124	-0.7863598	1.942394
	_cons	12.77188	5.164276	2.650085	22.89367

In the year before BMI measurement, the Gamma distribution is justified as an appropriate choice because β_1 =1.3199 is close to 1. Also, the 95% CI (0.467, 2.173) contains 1, reinforcing that Gamma is a reasonable choice. In the year after, while the Modified Park Test yielded a β1 coefficient of 0.578 (95% CI: -0.79 to 1.94), the results were statistically insignificant (p = 0.406), indicating uncertainty in variance structure. Given that the Gamma model demonstrated superior fit based on AIC and BIC compared to Poisson and Inverse Gaussian, we opted for the Gamma distribution as the most appropriate choice for modeling healthcare costs.

eTable 10: *AIC and BIC comparisons for models 1 to 3 – year before BMI measurement*

Model	N	df	AIC	BIC
Model 1: Unadjusted	268,231	10	4087885	4087990
Model 2: Adjusted (no interactions)	268,231	64	4056510	4057182
Model 3: Adjusted (Interactions)	268,212	362	4054076	4057877

Model 3, which included covariates and interaction terms, exhibited superior performance based on AIC compared to the unadjusted and partially adjusted models. However, Model 1 was favored according to BIC, reflecting its preference for model parsimony.

eTable 11: *AIC and BIC comparisons for models 1 to 3 – year after BMI measurement*

Model	N	df	AIC	BIC
Model 1: Unadjusted	268,231	10	4370652	4370757
Model 2: Adjusted (no interactions)	268,231	64	4349721	4350393
Model 3: Adjusted (Interactions)	267,435	354	4346872	4350587

Model 3, which included covariates and interaction terms, exhibited superior performance based on AIC compared to the unadjusted and partially adjusted models. However, Model 1 was favored according to BIC, reflecting its preference for model parsimony.

© 2025 Onyimadu O et al. *JAMA Network Open*.

eResults: Assessment of sample representativeness

To assess the representativeness of our analytic sample, we compared BMI category distributions in the CPRD-HES cohort with 2022/23 National Child Measurement Programme (NCMP) data for Reception (ages 4–5) and Year 6 (ages 10–11)[1], the only age groups with consistent national surveillance since 2006/07 (***eFigure 1*** and **eFigure 2**). BMI distributions in our cohort closely aligned with NCMP estimates at both ages (

© 2025 Onyimadu O et al. *JAMA Network Open*.

eTable 1), with only a modestly higher prevalence of obesity and underweight, which is expected in a primary care dataset where children have more frequent clinical contacts. These similarities indicate that, despite being derived from healthcare records, our sample broadly reflects national child weight distributions and supports the generalizability of our findings.

© 2025 Onyimadu O et al. *JAMA Network Open*.

eTable 12: *Predicted absolute annual total health care costs by BMI category, in the year before and after BMI measurement, from Model 3 (preferred covariate- and interaction-adjusted model).*

BMI Category	Year before BMI record Mean (£, SE)	95% CI (Lower–Upper)	Year after BMI record Mean (£, SE)	95% CI (Lower–Upper)
**Underweight**	1282.182 (45.40)	1193.206 – 1371.159	1516.358 (47.70)	1422.873 – 1609.844
**Healthy weight**	1286.234 (9.78)	1267.061 – 1305.406	1352.010 (9.95)	1332.513 – 1371.506
**Overweight**	1425.397 (42.98)	1341.152 – 1509.641	1418.855 (23.41)	1372.971 – 1464.740
**Obesity**	1399.142 (28.43)	1343.430 – 1454.854	1492.748 (36.18)	1421.833 – 1563.663
**Severe obesity**	1435.764 (47.12)	1343.403 – 1528.126	1541.589 (56.93)	1430.017 – 1653.162

SE = standard error; CI = confidence interval. Estimates derived from generalized linear/2-part models with robust SEs. To convert to 2022 US dollars, multiply by 1.44.

© 2025 Onyimadu O et al. *JAMA Network Open*.

eTable 13: *Marginal Difference in Predicted Total Healthcare Costs (GBP) Attributable to Underweight and Excess Weight Categories in the year before BMI measurement (Model 3), Clinical Practice Research Datalink (CPRD)-Hospital Episode Statistics (HES) electronic medical records (2014-2020)*

**Weight status**	**Incremental cost (£)**	**Lower 95% CI**	**Upper 95% CI**
Underweight	-25,407,625	-64,532,372	13,717,107
Overweight	171,425,536	74,845,325	268,005,888
Obesity (including severe obesity)	140,869,740	75,947,513	205,791,918
Excess weight	312,295,276	150,792,838	473,797,806

The marginal differences (or excess costs) in predicted total healthcare costs apply when children in the healthy weight category incur £0 in primary and secondary healthcare utilization costs.

eTable 14: *Marginal Difference in Predicted Total Healthcare Costs (GBP) Attributable to Underweight and Excess Weight Categories in the year after BMI measurement (Model 3), Clinical Practice Research Datalink (CPRD)-Hospital Episode Statistics (HES) electronic medical records (2014-2020)*

**Weight status**	**Incremental cost (£)**	**Lower 95% CI**	**Upper 95% CI**
Underweight	71,081,134	27,920,316	114,241,992
Overweight	84,890,266	20,450,624	149,329,920
Obesity (including severe obesity)	188,497,320	100,592,257	276,402,346
Excess weight	273,387,586	121,042,881	425,732,266

The marginal differences (or excess costs) in predicted total healthcare costs apply when children in the healthy weight category incur £0 in primary and secondary healthcare utilization costs.

eReference

1. NHS England. *National Child MeasurementProgramme, England, 2022/23 SchoolYear*. 2023. https://digital.nhs.uk/data-and-information/publications/statistical/national-child-measurement-programme/2022-23-school-year#

© 2025 Onyimadu O et al. *JAMA Network Open*.
