# Supplementary material for: Child Body Mass Index and Health Care Costs in England
Source: JAMA Netw Open. 2025 Oct 14;8(10):e2537560. doi: 10.1001/jamanetworkopen.2025.37560 (PMC12522009; doi:10.1001/jamanetworkopen.2025.37560)
Supplement: Supplement 2. — Data Sharing Statement [file jamanetwopen-e2537560-s002.pdf]

Data Sharing Statement

Onyimadu. Child Body Mass Index and Health Care Costs in England. *JAMA Netw Open*. Published October 14, 2025. doi:10.1001/jamanetworkopen.2025.37560

Data
Data available: No

Additional Information
Explanation for why data not available: Due to the confidential nature of the data, access is strictly controlled by the Clinical Practice Research Datalink (CPRD) obtained under license from the UK Medicines and Healthcare products Regulatory Agency (MHRA). The data are provided by patients and collected by the National Health Service (NHS) as part of their care and support. The CPRD protocol for this study can be found online here: https://www.cprd.com/approved-studies/economic-consequences-childhood-excess-weight-secondary-analyses-cprd
